# Effects of non-surgical periodontal therapy on gingival crevicular fluid CTRP-1, TNF-α, and IL-10 levels

**DOI:** 10.1007/s00784-025-06420-3

**Published:** 2025-06-21

**Authors:** Asena Kadayıf, Özge Elif Taşçi, Burcu Karaduman

**Affiliations:** 1 Faculty of Dentistry, Department of Periodontology, Biruni University, Merkezefendi, G/75 St. No: 1-13, Cevizlibağ/Istanbul, 34015 Türkiye; 2 Biruni University Research Center (BAMER), Biruni University, Istanbul, Türkiye

**Keywords:** CTRP-1, Periodontal disease, Gingival crevicular fluid, Non-surgical periodontal therapy, Biomarkers

## Abstract

**Objectives:**

This study investigated the gingival crevicular fluid (GCF) levels of complement-C1q tumor necrosis factor related protein-1 (CTRP-1), tumor necrosis factor-α (TNF-α), and interleukin-10 (IL-10) following non-surgical periodontal therapy (NSPT) in systemically healthy individuals with periodontal health (H), generalized gingivitis (G), and stage III grade B periodontitis (P). Moreover, this study aimed to investigate their diagnostic potential in distinguishing different periodontal diseases.

**Materials and methods:**

73 systemically healthy non-smoking individuals were divided into H (*n* = 25), G (*n* = 23) and P (*n* = 25) groups. Clinical periodontal parameters were recorded, and GCF samples were collected at baseline in all groups, and 3rd month after NSPT in the G and P groups. GCF levels of CTRP-1, TNF-α, and IL-10 were analyzed using ELISA. The area under the curve (AUC) was assessed using the receiver operating characteristic curve analysis.

**Results:**

CTRP-1 levels were significantly elevated in the G and P group compared to the H group (*p* < 0.001), while there was no significant difference between the G and P groups (*p* = 0.095). TNF-α and IL-10 levels were significantly higher in the P group compared to the other groups (*p* < 0.001). GCF CTRP-1 demonstrated an excellent diagnostic performance to discriminate periodontitis and gingivitis from periodontal health (AUC value of 0.998 and 0.974 with 100% and 91.3% sensitivity).

**Conclusion:**

CTRP-1 could serve as a potential biomarker for periodontal diseases. CTRP-1 demonstrated excellent diagnostic performance in distinguishing stage III grade B periodontitis and gingivitis from periodontal health.

**Clinical relevance:**

GCF CTRP-1 levels may serve as a valuable marker for diagnosing periodontal diseases.

## Introduction

Periodontal diseases are chronic, multifactorial inflammatory conditions characterized by an imbalance between the host immune response and microbial dysbiosis within the plaque biofilm. This dysregulated interaction promotes disease progression, ultimately leading to periodontal pocket formation, connective tissue degradation, and alveolar bone loss [1, 2]. Emerging evidence suggests that systemic inflammatory mediators contribute to periodontal disease pathogenesis, emphasizing the need to explore specific biomarkers that could aid in early diagnosis and prognosis [3].

The adipose tissue is defined as the largest endocrine organ that secretes adipose-derived hormones (adipokines) including adiponectin, leptin, resistin, visfatin, tumor necrosis factor-alpha (TNF-α) and interleukin (IL)-6. Adipokines play a crucial role in regulating metabolic activities and immune responses, and they contribute to the development of various systemic conditions such as hypertension, diabetes, metabolic syndrome, and cardiovascular diseases [4]. Recent studies have demonstrated a significant association between adiponectin and periodontitis. In particular, adiponectin receptor expression in periodontal tissues has been found to be lower in patients with periodontitis compared to healthy individuals [5]. Moreover, the concentration of adiponectin in gingival crevicular fluid (GCF) has been shown to increase following non-surgical periodontal therapy (NSPT), supporting its anti-inflammatory role [6]. In addition to adiponectin, other adipokines, such as leptin, resistin, and visfatin, have also been implicated in the pathogenesis of periodontal disease [7–9]. These findings suggest a significant interaction between adipose tissue-derived mediators and periodontal inflammation and their potential diagnostic and therapeutic implications warrant further investigation.

Complement-C1q/tumor necrosis factor related protein-1 (CTRP-1), an adiponectin paralog family member, has emerged as a significant regulator of immune and metabolic homeostasis [10]. CTRP-1 has been shown to modulate the production of primary pro-inflammatory cytokines such as TNF-α and IL-6, thereby suggesting its involvement in the mechanisms underlying inflammatory diseases [11, 12]. Recent studies indicate that CTRP-1 may act as an immune modulator, balancing both pro-inflammatory and anti-inflammatory pathways [11, 12]. This dual role has sparked interest in its potential as a biomarker for inflammatory diseases. Clinical studies have demonstrated that serum CTRP-1 levels are significantly elevated in individuals with type II diabetes and positively correlated with insulin secretion [13]. Similarly, increased serum CTRP-1 concentrations have been observed in patients with atherosclerosis [14]. Despite these findings, the precise mechanism through which CTRP-1 influences inflammation and metabolic dysfunction remains unclear. Therefore, more research is needed to elucidate its regulatory functions and explore its potential as a therapeutic target for inflammatory diseases, including periodontal diseases, as well as metabolic disorders.

TNF-α is a pro-inflammatory mediator in periodontal tissue destruction that promotes osteoclastogenesis, induces matrix metalloproteinases (MMPs), and enhances inflammatory cell infiltration, leading to extracellular matrix degradation and alveolar bone resorption [2, 15]. Elevated TNF-α levels are strongly associated with periodontal disease severity [15]. Additionally, TNF-α has a crucial role in the upregulation of other inflammatory mediators, further enhancing periodontal tissue degradation [16]. Kim et al. [17] showed that lipopolysaccharide (LPS) administration increased CTRP-1 expression following TNF-α and IL-1β induction, suggesting that CTRP-1 may function as a component of the inflammatory cascade.

IL-10 is an anti-inflammatory cytokine related in the regulation of immune responses and the maintenance of tissue homeostasis by limiting excessive inflammatory reactions [18, 19]. IL-10 downregulates the release of pro-inflammatory mediators, including TNF-α, IL-1β, and IL-6, thereby limiting tissue destruction and promoting periodontal wound healing [18–20].

**Fig. 1:**
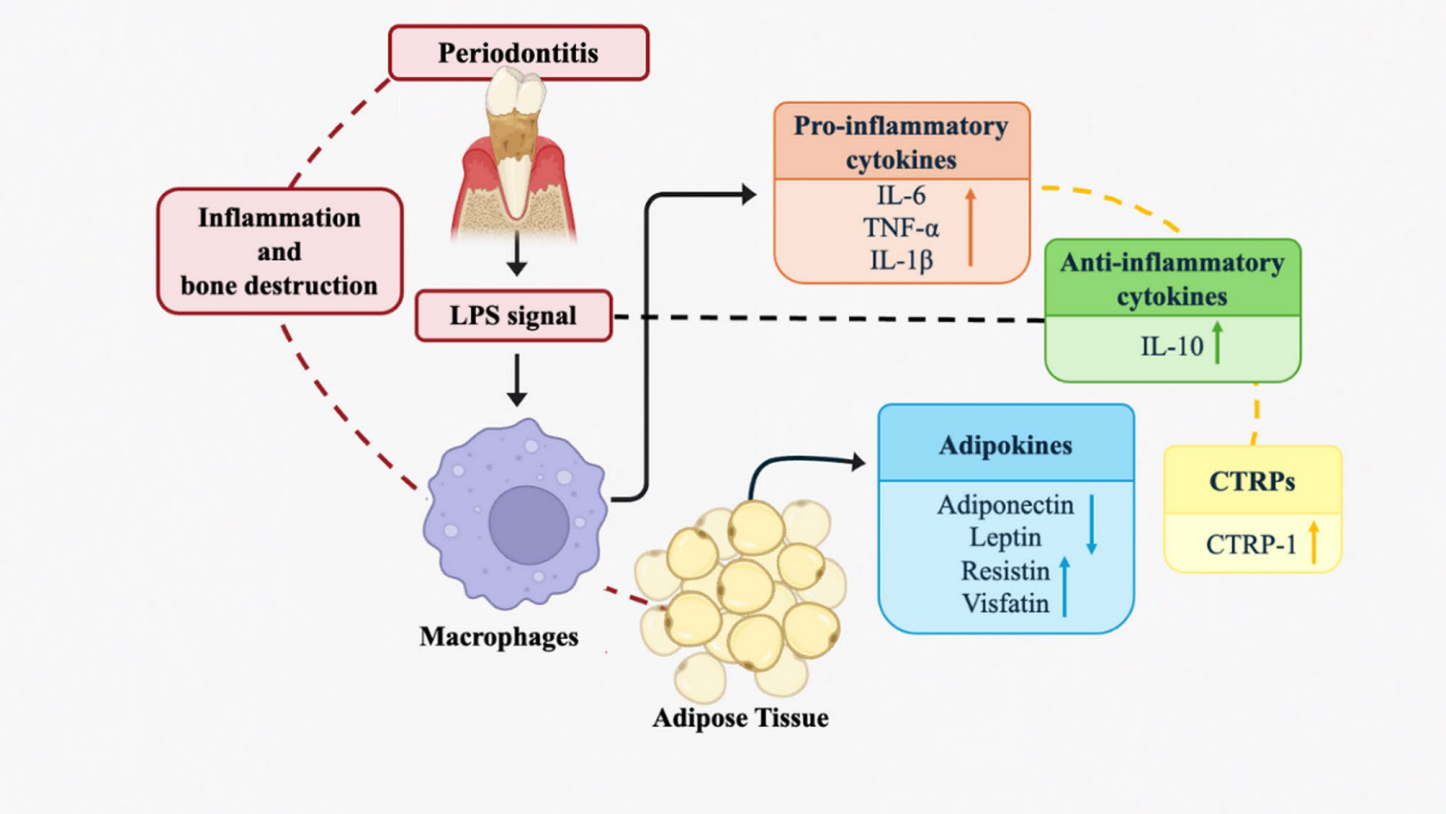
Schematic illustration of the relationship between periodontal inflammation and adipose tissue-derived cytokines and adipokines. Abbreviations: LPS: Lipopolysaccharide; IL-6: Interleukin-6; TNF-α: Tumor necrosis factor-alpha; IL-1β: Interleukin-1 beta; IL-10: Interleukin-10; CTRP: Complement-C1q tumor necrosis factor related protein. This schematic figure was created using Biorender (Biorender.com) and finalized by the authors

According to our current knowledge, the evaluation of GCF CTRP-1 levels in different periodontal conditions has not yet been reported. Considering the modulatory role of CTRP-1 in inflammation, analyzing its association with inflammatory mediators like TNF-α and IL-10 in periodontal disease may offer important perspectives on disease progression and potential interventions. Therefore, this study focused to explore the relationship between CTRP-1, TNF-α, and IL-10 levels, and periodontal inflammation by comparing their levels in GCF samples from systemically healthy individuals with periodontal health, gingivitis, and Stage III Grade B (SIIIGB) periodontitis, as well as to evaluate the effects of NSPT on these biomarkers. A schematic representation of the proposed interactions among CTRP-1, TNF-α, and IL-10 in periodontal inflammation is presented in Fig. 1. The null hypothesis of this study was that there would be no significant difference in the levels of CTRP-1, TNF-α, and IL-10 in GCF among systemically healthy individuals with periodontal health, gingivitis, and SIIIGB periodontitis, and NSPT would have no significant effect on biomarker levels.

## Materials and methods

### Study population and clinical evaluations

**Fig. 2:**
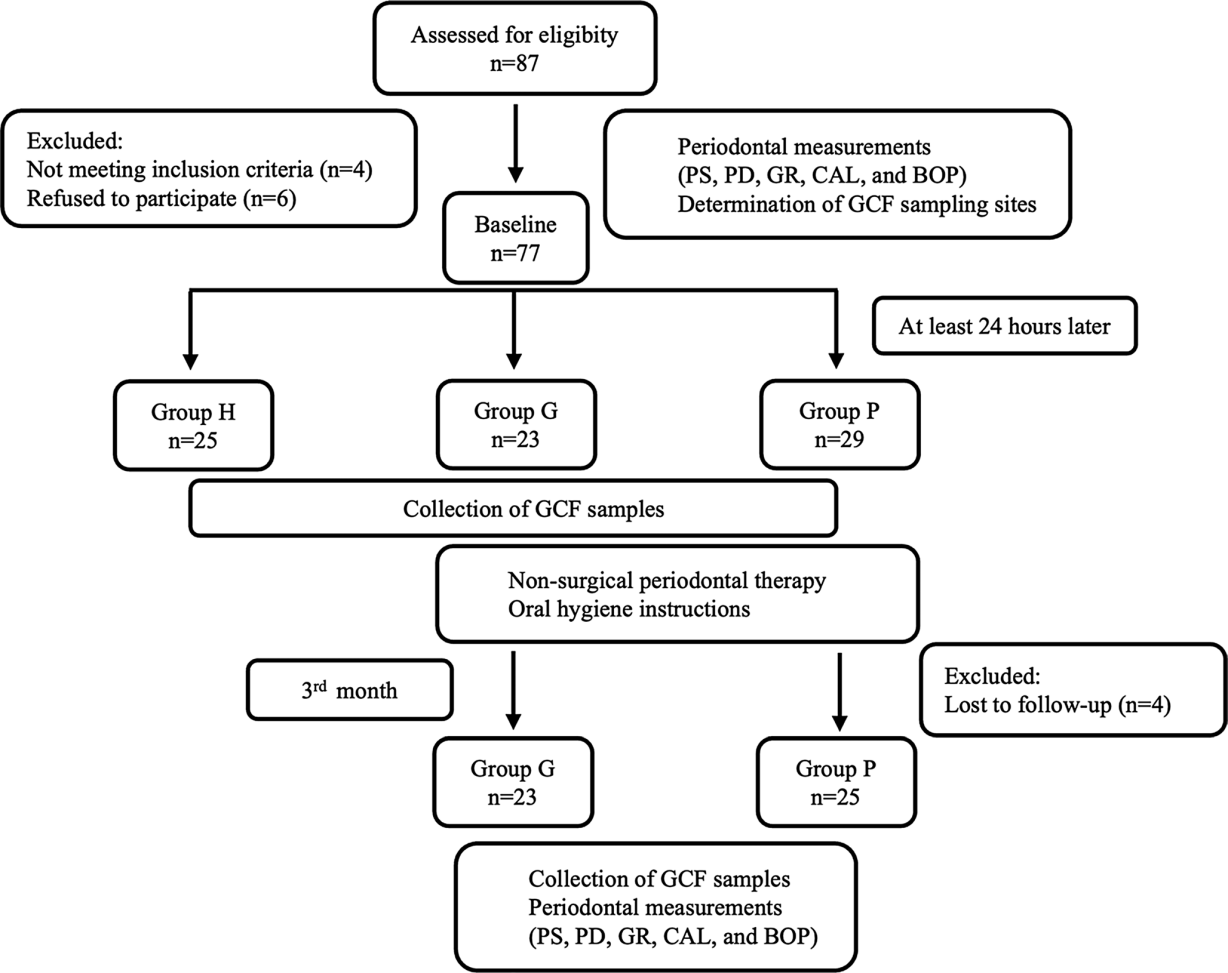
Flowchart of the study. Abbreviations: PS: plaque score; PD: probing depth; GR: gingival recession; CAL: clinical attachment level; BOP: bleeding on probing; GCF: gingival crevicular fluid; Group P: stage III grade B periodontitis; Group G: gingivitis; Group H: periodontal health

A total of 87 participants (48 females and 39 males) applied to the Department of Periodontology, Faculty of Dentistry, Biruni University, Istanbul, Türkiye between October 2023 and September 2024; and the study was completed with 73 participants. A flowchart outlining the research design and participant allocation is presented in Fig. 2. This study was approved by Biruni University Clinical Research Ethics Committee (Decision number: 2015-KAEK-79-23-07) and was conducted in accordance with Declaration of Helsinki. The study protocol was submitted at ClinicalTrials.gov (NCT06175624).

Medical and dental histories, panoramic radiographs, intraoral photographs, and informed consent forms were acquired from all participants. The inclusion criteria were as follows: age > 18 years, systemically healthy (self-reported), non-smoker (including cigarettes and vaping), having at least 20 teeth (excluding third molars), having at least one periodontal site with a probing depth (PD) ≥ 5 mm in each quadrant, and a body mass index (BMI) ≤ 29.9 kg/m². The exclusion criteria were as follows: receiving periodontal treatment within the last 6 months, using any medication within the past 6 months, including immunosuppressants, corticosteroids, anti-inflammatory and anti-resorptive agents, antibiotics, antiepileptics, antihypertensives, anticoagulants, hormonal contraceptives, or nutritional supplements, and being a chronic smoker or regular alcohol consumer. Pregnant or nursing participants were excluded from the study.

The 2017 World Workshop guidelines on periodontal and peri-implant disease classification were used to evaluate periodontal status [1]. The participants were categorized into the following three groups:

- **Periodontal Healthy (Group H; *n* = 25)**: PD ≤ 3 mm, absence of clinical periodontal inflammation and bleeding on probing (BOP) [21] ≤ 10%, no clinical attachment loss, no radiological bone loss and no history of periodontal disease [22].
- **Plaque-induced Generalized Gingivitis (Group G; *n* = 23**): PD ≤ 3 mm and BOP ≥ 30%, no clinical attachment loss, and no radiological bone loss [23].
- **Stage III Grade B (SIIIGB) Periodontitis (Group P; *n* = 25)**: at least 2 non-adjacent teeth clinical attachment level (CAL) ≥ 5 mm, radiographic bone loss extending to apical third, loss of ≤ 4 teeth due to periodontal disease, PD ≥ 6 mm, vertical bone loss ≥ 3 mm, moderate rate of progression (Grade B) [24].

Clinical measurements were recorded by a blinded and calibrated investigator (A.K.) using a UNC 15 periodontal probe (University of North Carolina, Hu-Friedy Ins. Co, USA) at six sites per tooth (mesiobuccal, midbuccal, distobuccal, mesiolingual, midlingual, distolingual), except the third molars. To assess intra-examiner reliability, PD values were recorded at one-day intervals in five healthy individuals and five SIIIGB periodontitis patients who were not included in the study. The intraclass correlation coefficient (ICC) for PD was 0.995 (0.982-0.999), indicating high measurement consistency. The clinical parameters evaluated in this study included plaque score (PS), PD, gingival recession (GR), CAL, and BOP. Clinical periodontal parameters were recorded, and GCF samples were collected at baseline in all groups, as well as at the 3rd month following NSPT in the G and P groups. All clinical measurements were performed at least 24 h prior to sample collection.

### Collection of GCF samples

GCF samples were collected between 9 and 11 a.m. to standardize the daily circadian volume variations. One GCF sample was obtained from each quadrant of every patient in all groups, resulting in a total of four samples per patient. The selection of sampling sites was based on specific clinical criteria for each group [20, 25, 26]:

Group P: Samples were collected from the deepest periodontal pockets (PD ≥ 5 mm).

Group G: Sites with visible clinical inflammation and BOP (+) were selected.

Group H: Sampling areas included gingival sulcus without visible inflammation and BOP (−) sites.

Sampling sites were isolated with cotton rolls to prevent saliva and blood contamination. The supragingival plaque was carefully removed with a scaler (Hu-Friedy, Chicago, IL, USA) without irritating the gingiva, and the tooth surfaces were dried using an air spray. Sterilized standard-sized paper strips (Periopaper, ProFlow Inc., Amityville, NY, USA) were placed 1–2 mm into the gingival sulcus for 30 s using the intracrevicular technique. Paper strips contaminated with blood, saliva, or plaque were not included to ensure accurate GCF volume measurement. The collected GCF volume was measured using a pre-calibrated Periotron 8010™ (Oroflow, NY, USA) and the Periotron scores were converted to µL based on a standard curve. After measurement, all paper strips were pooled in Eppendorf tubes and stored at -80 °C until the day of analysis.

### Non-surgical periodontal therapy (NSPT)

Immediately following periodontal examination and sample collection, NSPT was performed by an experienced investigator (Ö.E.T.). Oral hygiene instructions were provided to all patients. The treatment protocol followed the sequential steps outlined in the European Federation of Periodontology (EFP) S3 Level Clinical Practice Guideline [27]. The first step consisted of supragingival instrumentation, involving the removal of the supragingival biofilm and calculus using both hand instruments (Hu-Friedy, Chicago, IL, USA) and an ultrasonic device (EMS Minimaster, Nyon, Switzerland). The second step of treatment consisted of subgingival instrumentation, aiming to eliminate the subgingival biofilm and calculus with the use of the same hand instruments and ultrasonic device. The first step of periodontal treatment was applied in both the G and P groups, while the second step of periodontal treatment was applied only to the P group one week after the first step. Participants in the H group did not receive NSPT, as they presented with clinically healthy periodontal tissues. No additional antimicrobial agents or medications, including mouth rinses, were administered. Patients were also specifically instructed not to use any antimicrobial mouth rinses during the study period.

### Biochemical analysis

Biochemical analyses were conducted after all the collection of all samples was completed. GCF levels of CTRP-1, TNF-α, and IL-10 were evaluated by Enzyme-Linked Immunosorbent Assay (ELISA) method using human ELISA kits (Sunred Biotechnology, Shanghai, China). The total amount of cytokines was calculated. According to the manufacturer’s instructions, the CTRP-1 sensitivity level was 0.085 ng/ml, the TNF-α sensitivity level was 2.827 ng/l, and the IL-10 sensitivity level was 9.012 pg/ml, respectively.

Prior to analysis, GCF samples were allowed to equilibrate to room temperature and gently vortexed to ensure homogeneity. A total of 100 µL phosphate-buffered saline (PBS) was added to each sample and incubated for 30 min at room temperature. For each well, 50 µL of standards and 40 µL of each sample were pipetted. Subsequently, 10 µL of the appropriate biotin-conjugated antibody solution was added to each sample well, excluding the standard wells. The plates were covered and incubated at 37 °C for 60 min. Following incubation, the plates were washed thoroughly 5 times with wash buffer. After drying, 50 µL of chromogen A and 50 µL of chromogen B were added to each well. The plates were then incubated at 37 °C for an additional 15 min. The reaction was terminated by adding 50 µL of stop solution, resulting in a color change from blue to yellow. The optical density of each well was measured at 450 nm using a microplate reader (Diagnostic Automation Inc., USA). Based on the standard curve, the total amount of each cytokine in the samples was calculated.

### Statistical analysis

The sample size was calculated using the G-Power program (Version 3.1.9.2, Heinrich Heine University, Düsseldorf, Germany). To detect a statistical difference with a minimum effect size of 0.4 (f-type), a power of 90%, and a significance level of α = 0.05, at least 63 participants were required. Considering a potential loss to follow-up rate of 10%, the final sample size was set to *n* = 69, with *n* = 23 allocated to each group.

Data distribution was examined using the Shapiro-Wilk test. The Mann–Whitney U test was used to compare two independent groups that did not follow a normal distribution. The Kruskal-Wallis test (K-W) was applied to compare the three groups. Post-hoc comparisons for variables showing significance in the K-W test were performed using Dunn’s test. Wilcoxon test was used to compare two dependent groups that did not follow a normal distribution. The Fisher-Freeman Halton test was used to compare categorical variables between groups. The ability of biochemical variables to distinguish between patients and healthy controls was evaluated using Receiver Operating Characteristic (ROC) curve analysis. The cut-off value was determined based on Youden’s J index. All statistical analyses were executed using a software program (SPSS v.20.0, IBM, Chicago, IL, USA) with a significance level of 0.05, with a 95% confidence interval.

## Results

### The study population and clinical measurements

Table 1 presents the distribution of age, sex, BMI, and the number of teeth in the study groups and a comparison of full-mouth clinical parameters between the groups. The P group had a higher age and BMI than the H (*p* < 0.001) and G (*p* = 0.022) groups. The sex distribution between the groups was similar (*p* = 0.429). The number of teeth was significantly lower in the P group than in the G (*p* = 0.005) and H (*p* = 0.010) groups. The baseline BOP and PS values were lower in the H group than in the G (*p* < 0.001) and P groups (*p* < 0.001). PD and CAL values were significantly higher in the P group than in the G (*p* < 0.001) and H (*p* < 0.001) groups and significantly higher in the G group than in the H group (*p* < 0.020). PS, PD, CAL, and BOP values were significantly higher in the G (*p* < 0.001, all parameters) and P (*p* < 0.001, all parameters) groups at baseline than at 3rd month. The GR was significantly higher in the P group at the 3rd month (*p* = 0.001).

**Table 1:** Comparisons of age, sex, body mass index and full mouth clinical parameters among groups at baseline and 3rd month

Full Mouth								
Variables	Time points	Group *P*^1^ *n* = 25	Group G^2^ *n* = 23	Group H^3^ *n* = 25	(1-2-3) *p*	(1–2) *p*	(1–3) *p*	(2–3) *p*
Age (years)		42 (24–58)	30 (21–45)	28 (19–57)	**< 0.001** ^*^	**0.001** ^**a**^	**< 0.001** ^**a**^	1^a^
Sex n (%)					0.429^d^			
Female		17(%68)	12(%52.2)	17 (%68)				
Male		8 (%32)	11(%47.8)	8 (%32)				
BMI (kg/m^2^)		27.30 (20.50–29.60)	23.70 (19.30–28.60)	21.50 (18.40–29.20)	**< 0.001** ^*^	**0.022** ^**a**^	**< 0.001** ^**a**^	0.387^a^
Number of Teeth	Baseline	25 (20–28)	28(24–28)	28 (22–28)	**0.002** ^*^	**0.005** ^**a**^	**0.010** ^**a**^	1^a^
	3rd month	25 (20–28)	27 (24–28)	-		**0.001** ^**b**^		
	p	**0.033** ^**c**^	0.157^c^	-				
	Δ0–3	0 (-2:1)	0 (-1:0)	-		0.171^b^		
PS (%)	Baseline	62.10 (18.05–100)	63.40 (10–100)	5.80 (0-35.70)	**< 0.001** ^*^	1^a^	**< 0.001** ^**a**^	**< 0.001** ^**a**^
	3rd month	15 (1–40)	13 (5-27.10)	-		0.148^b^		
	p	**< 0.001** ^**c**^	**< 0.001** ^**c**^	-				
	Δ0–3	-53.80 (-73:-10.05)	-52.50 (-84:-3)	-		0.451^b^		
PD (mm)	Baseline	3.90 (3.10–5.20)	2.30 (2.10–2.70)	2 (1.60–2.50)	**< 0.001** ^*^	**< 0.001** ^**a**^	**< 0.001** ^**a**^	**0.020** ^**a**^
	3rd month	3.10 (2.50–4.10)	2 (1.90–2.40)	-		**< 0.001** ^**b**^		
	p	**< 0.001** ^**c**^	**< 0.001** ^**c**^	-				
	Δ0–3	-0.90 (-1.90:0.20)	-0.20 (-0.40:-0.10)	-		**< 0.001** ^**b**^		
GR (mm)	Baseline	0.06 (0-0.42)	-	-	NA			
	3rd month	0.17 (0-1.18)	-	-		NA		
	p	**0.001** ^**c**^	-	-				
	Δ0–3	0.10 (-0.09:1.15)	-	-		NA		
CAL (mm)	Baseline	4.10 (3.20–5.20)	2.30 (2.10–2.70)	2 (1.60–2.50)	**< 0.001** ^*^	**< 0.001** ^**a**^	**< 0.001** ^**a**^	**0.020** ^**a**^
	3rd month	3.40 (2.70–4.70)	2 (1.90–2.40)	-		**< 0.001** ^**b**^		
	p	**< 0.001** ^**c**^	**< 0.001** ^**c**^	-				
	Δ0–3	-0.80 (-1.50:0.50)	-0.20 (-0.40:-0.10)	-		**< 0.001** ^**b**^		
BOP (%)	Baseline	80.30 (53.10–100)	78.80 (49–100)	5.80 (0-9.50)	**< 0.001** ^*^	1^a^	**< 0.001** ^**a**^	**< 0.001** ^**a**^
	3rd month	28 (3–54)	14.50 (5-28.50)	-		**< 0.001** ^**b**^		
	p	**< 0.001** ^**c**^	**< 0.001** ^**c**^	-				
	Δ0–3	-56 (-74:-4.50)	-60.90(-88:-36.50)	-		**0.045** ^**b**^		

Abbreviations: P group: Stage III Grade B Periodontitis; G group: gingivitis; H group: periodontal health; BMI: body mass index; GCF: gingival crevicular fluid; PS: plaque score; PD: probing depth; GR: gingival recession; CAL: clinical attachment level; BOP: bleeding on probing. Δ0–3: The change between baseline and 3 months. Data are presented as median (minimum-maximum) values, except for sex, which is shown as number (%). *Kruskal-Wallis test; a: Dunn’s test; b: Mann-Whitney U test; c: Wilcoxon test; d: Fisher-Freeman-Halton test; NA: not applicable. ***p* < 0.05 statistical differences are marked in bold**

Table 2 presents the clinical parameters associated with the sampling sites. At baseline, the GCF volume was significantly greater in the P group compared to the G (*p* = 0.018) and H (*p* < 0.001) groups. Additionally, the G group exhibited a higher GCF volume than the H group (*p* < 0.001) at baseline. The H group exhibited significantly lower PS and BOP values compared to both the G (*p* < 0.001 and *p* < 0.001, respectively) and the P (*p* < 0.001 and *p* < 0.001, respectively) groups. The PD was significantly higher in the P group than in both the G (*p* < 0.001) and H (*p* < 0.001) groups. Furthermore, the G group exhibited a significantly greater PD value compared to the H group (*p* = 0.004).

**Table 2:** Comparisons of clinical parameters of the sampling sites among groups at baseline and 3rd month

Sampling Sites								
Variables	Time points	Group *P*^1^ (*n* = 25)	Group G^2^ (*n* = 23)	Group H^3^ (*n* = 25)	(1-2-3) *p*	(1–2) *p*	(1–3) *p*	(2–3) *p*
GCF (µL)	Baseline	2.59 (0.32–4.15)	1.90 (1.51–2.75)	1.25 (0.62–1.90)	**< 0.001** ^*^	**0.018** ^**a**^	**< 0.001** ^**a**^	**< 0.001** ^**a**^
	3rd month	1.46 (0.77–1.87)	1.11 (0.80–1.82)	-		**< 0.001** ^**b**^		
	p	**< 0.001** ^**c**^	0.157^c^	-				
	Δ0–3	-1.27 (-2.38:1.41)	-0.67 (-1.78:-0.33)	-		**0.004**		
PS (%)	Baseline	75 (0-100)	75 (0-100)	0 (0–25)	**< 0.001** ^*^	1^a^	**< 0.001** ^**a**^	**< 0.001** ^**a**^
	3rd month	0 (0–75)	0 (0–25)	-		0.920^b^		
	p	**< 0.001** ^**c**^	**< 0.001** ^**c**^	-				
	Δ0–3	-50 (-100:25)	-75 (-100:0)	-		0.615^b^		
PD (mm)	Baseline	6 (3-7.50)	2.50 (2-3.25)	2 (1-2.25)	**< 0.001** ^*^	**< 0.001** ^**a**^	**< 0.001** ^**a**^	**0.004** ^**a**^
	3rd month	3.50 (2-6.25)	2 (1.75–2.50)	-		**< 0.001** ^**b**^		
	p	**< 0.001** ^**c**^	**< 0.001** ^**c**^	-				
	Δ0–3	-2.50 (-4.25:0.75)	-0.25 (-1:0)	-		**< 0.001** ^**b**^		
GR (mm)	Baseline	0 (0–1)	-	-	NA			
	3rd month	0.25 (0–2)	-	-		NA		
	p	**< 0.001** ^**c**^	-	-				
	Δ0–3	0.25 (-1:1.25)	-	-		NA		
CAL (mm)	Baseline	6.37 (3.50–8.50)	2.50 (2-3.25)	2 (1-2.25)	**< 0.001** ^*^	**< 0.001** ^**a**^	**< 0.001** ^**a**^	**< 0.001** ^**a**^
	3rd month	4 (2–7)	2 (1.75–2.50)	-		**< 0.001** ^**b**^		
	p	**< 0.001** ^**c**^	**< 0.001** ^**c**^	-				
	Δ0–3	-2.50 (-5:0.50)	-0.25 (-1:0)	-		**< 0.001** ^**b**^		
BOP (%)	Baseline	100 (100–100)	100 (100–100)	0 (0–0)	**< 0.001** ^*^	1^a^	**< 0.001** ^**a**^	**< 0.001** ^**a**^
	3rd month	25 (0–75)	0 (0–25)	-		**< 0.001** ^**b**^		
	p	**< 0.001** ^**c**^	**< 0.001** ^**c**^	-				
	Δ0–3	-75 (-100:-25)	-100 (-100:75)	-		**< 0.001** ^**b**^		

Abbreviations: P group: Stage III Grade B Periodontitis; G group: gingivitis; H group: periodontal health; BMI: body mass index; GCF: gingival crevicular fluid; PS: plaque score; PD: probing depth; GR: gingival recession; CAL: clinical attachment level; BOP: bleeding on probing. Δ0–3: The change between baseline and 3 months. Data are presented as median (minimum-maximum) values. *Kruskal-Wallis test; a: Dunn’s test; b: Mann-Whitney U test; c: Wilcoxon test; NA: not applicable. ***p* < 0.05 statistical differences are marked in bold**

At the 3-month, the reduction in GCF volume was significantly greater in the P group (*p* < 0.001), whereas in the G group, no statistically significant difference was observed (*p* = 0.157). At 3 month, the values for PS, PD, and BOP in both the G and P groups exhibited a statistically significant reduction compared to their baseline measurements (*p* < 0.001, all parameters). However, there was no significant difference in PS between the groups at 3 months. In contrast, PD, CAL, and BOP values remained significantly higher in the P group compared to the G group (*p* < 0.001, for all parameters).

### Biochemical results

The biochemical results are presented in Table 3. At baseline, the GCF CTRP-1 levels were significantly lower in the H group than in the G (*p* < 0.001) and P (*p* < 0.001) groups, although there was no significant difference between the G and P groups at baseline (*p* = 0.095). At the 3rd month, CTRP-1 levels significantly decreased in both the P (*p* < 0.001) and G (*p* < 0.001) groups; however, the difference between these groups was not statistically different (*p* = 0.170). At baseline, the levels of GCF TNF-α and IL-10 were significantly elevated in the P group compared to the G (*p* = 0.020) and H (*p* < 0.001) groups, and in the G group compared to the H group (*p* < 0.001). At 3rd month, GCF TNF-α levels exhibited a significant reduction in both the P (*p* < 0.001) and G (*p* < 0.001) groups; however, the levels remained elevated in the P group (*p* < 0.001). At 3 month, GCF IL-10 levels remained significantly elevated in the P group compared to those in the G group (*p* < 0.016). Additionally, the change in IL-10 levels in the GCF was significantly greater in the P group (*p* < 0.001).

**Table 3:** Inter- and intra-group comparison of GCF biochemical parameters at baseline and 3rd month

Parameters	Time points	Group *P*^1^ (*n* = 25)	Group G^2^ (*n* = 23)	Group H^3^ (*n* = 25)	(1-2-3) *p*	(1–2) *p*	(1–3) *p*	(2–3) *p*
GCF CTRP-1 (pg/30s)	Baseline	22 (14.31–33.75)	17.82 (12.61–24.24)	10.49 (4.73–14.94)	**< 0.001** ^*^	0.095^a^	**< 0.001** ^**a**^	**< 0.001** ^**a**^
	3rd month	11.86 (5.48–18.25)	9.41 (6.86–14.36)	-		0.861^b^		
	p	**< 0.001** ^**c**^	**< 0.001** ^**c**^	-				
	Δ0–3	-9.70 (-24.99:0.36)	-8.38 (-14.13:-3.97)	-		0.170^b^		
GCF TNF-α (ng/30s)	Baseline	0.63 (0.38–1.07)	0.46 (0.23–0.65)	0.26 (0.15–0.32)	**< 0.001** ^*^	**0.020** ^**a**^	**< 0.001** ^**a**^	**< 0.001** ^**a**^
	3rd month	0.34 (0.20–0.49)	0.24 (0.14–0.31)	-		**< 0.001** ^**b**^		
	p	**< 0.001** ^**c**^	**< 0.001** ^**c**^	-				
	Δ0–3	-0.33 (-0.69:0.01)	-0.23 (-0.40:0.02)	-		**0.044** ^**b**^		
GCF IL-10 (ng/30s)	Baseline	2.68 (1.57–3.56)	1.81 (1.25–2.68)	0.88 (0.42–1.14)	**< 0.001** ^*^	**0.004** ^**a**^	**< 0.001** ^**a**^	**< 0.001** ^**a**^
	3rd month	1.26 (0.63–1.77)	1.06 (0.64–1.55)	-		**0.016** ^**b**^		
	p	**< 0.001** ^**c**^	**< 0.001** ^**c**^	-				
	Δ0–3	-1.48 (-2.25:-0.11)	-0.78 (-1.91:-0.13)	-		**< 0.001** ^**b**^		

Abbreviations: Group P: stage III grade B periodontitis; Group G: gingivitis; Group H: periodontal health; GCF: gingival crevicular fluid; CTRP-1: Complement-C1q tumor necrosis factor related protein-1; TNF-α: Tumor necrosis factor-alpha; IL-10: Interleukin-10; ng, nanogram; pg: picogram, Δ0–3: The change between baseline and 3 months. Data are presented as median (minimum-maximum) values. *Kruskal-Wallis test; a: Dunn’s test; b: Mann-Whitney U test; c: Wilcoxon test; NA: not applicable. ***p* < 0.05 statistical differences are marked in bold**

Table 4 presents the relationships between biochemical parameters and study groups. To account for possible confounding variables such as age and BMI, logistic regression analyses were utilized. The healthy group served as the reference group. In both unadjusted and age-adjusted analyses, significant associations were identified between SIIIGB and GCF levels of CTRP-1, TNF-α, and IL-10 (*p* < 0.001, *p* < 0.001, and *p* < 0.001, respectively). Additionally, gingivitis was significantly associated with CTRP-1 and TNF-α (*p* = 0.004 and *p* < 0.001, respectively). Similarly, in both unadjusted and BMI-adjusted analyses, significant associations were identified between SIIIGB and GCF levels of CTRP-1 (*p* < 0.001, *p* < 0.001), TNF-α (*p* < 0.001, *p* < 0.001), IL-10 (*p* < 0.001, *p* < 0.001), as well as between gingivitis and CTRP-1 (*p* = 0.004, *p* = 0.005), and TNF-α (*p* < 0.001, *p* = 0.001).

**Table 4:** Logistic regression analysis of GCF biomarkers in SIIIGB periodontitis and gingivitis groups

	Periodontitis SIIIGB vs. Periodontal Health			Gingivitis vs. Periodontal Health		
	Unadjusted OR (95% CI)	Adjusted (Age) OR (95% CI)	Adjusted (BMI) OR (95% CI)	Unadjusted OR (95% CI)	Adjusted (Age) OR (95% CI)	Adjusted (BMI) OR (95% CI)
GCF CTRP-1	5.11 (2.012–13.006)	5.364 (1.993–14.383)	4.834 (1.939–12.048)	3.793 (1.530–9.405)	4.107 (1.564–10.782)	3.571 (1.468–8.691)
	**< 0.001**	**< 0.001**	**0.001**	**0.004**	**0.004**	**0.005**
GCF TNF-α	5.39 (1.541–2.882)	2.976 (4.612–27.116)	1.794 (5.044–6.379)	6.60 (7.854–21.553)	7.153 (9.762–21.524)	7.646 (7.777–8.520)
	**< 0.001**	**< 0.001**	**< 0.001**	**< 0.001**	**< 0.001**	**0.001**
GCF IL-10	0.273 (0.266–0.279)	0.874 (0.214–0.356)	0.817 (0.633–0.756)	-	-	-
	**< 0.001**	**< 0.001**	**< 0.001**			

Abbreviations: SIIIGB: Stage III Grade B; 95% CI: confidence interval of 95%; OR: odds ratio; GCF: gingival crevicular fluid; CTRP-1: Complement-C1q tumor necrosis factor related protein-1; TNF-α: Tumor necrosis factor-alpha; IL-10: Interleukin-10. ***p* < 0.05 statistical differences are marked in bold**

**Fig. 3:**
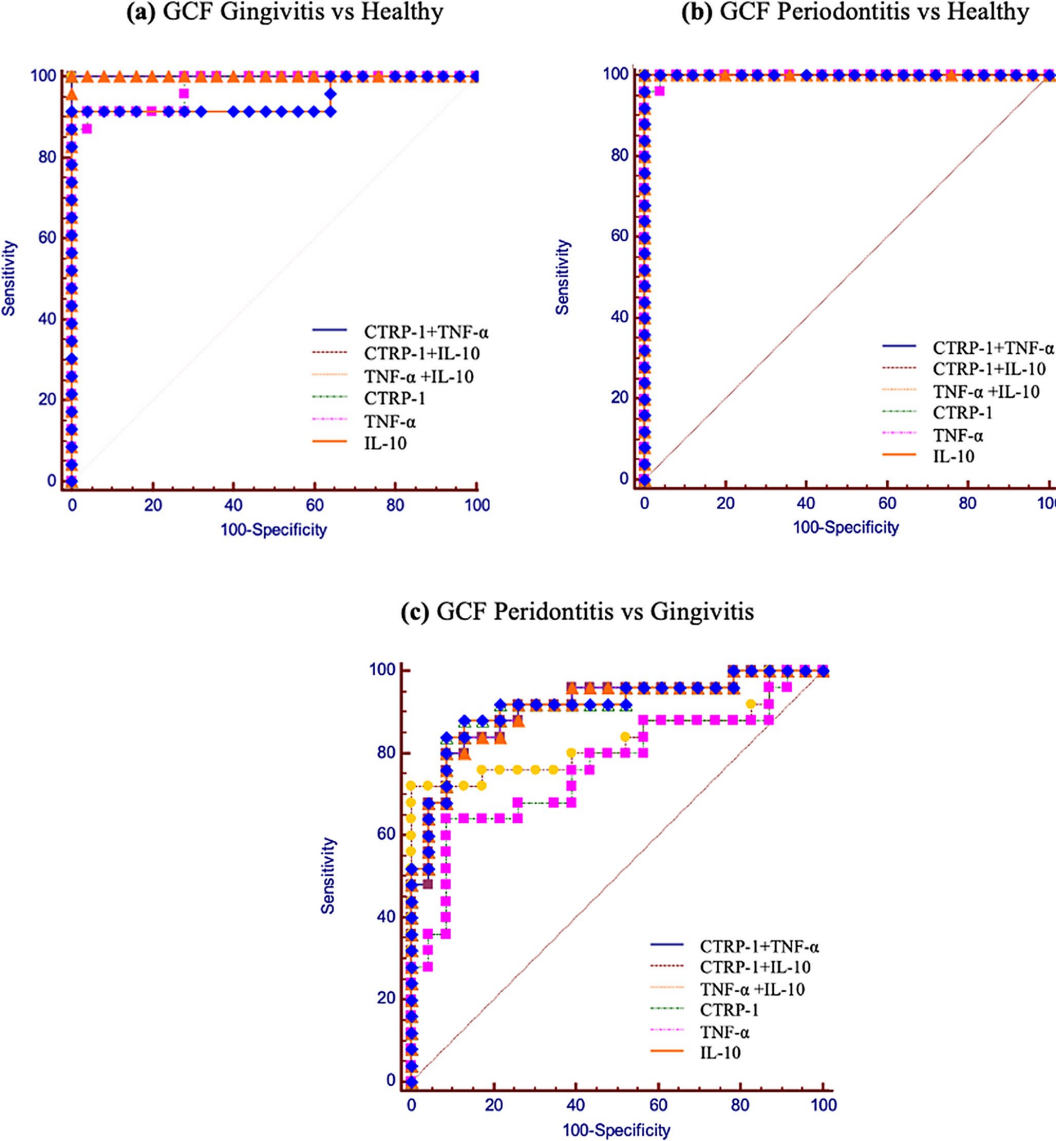
ROC curve analysis for GCF biomarkers and their combinations: (**a**) discriminate gingivitis patients from healthy individuals; (**b**) discriminate periodontitis patients from healthy individuals; (**c**) discriminate periodontitis patients from gingivitis patient. Abbreviations: CTRP-1: Complement-C1q tumor necrosis factor related protein-1; TNF-α: Tumor necrosis factor-alpha; IL-10: Interleukin-10. The cut-off values were calculated according to the Youden’s J index

Table 5; Fig. 3 display the AUC, sensitivity, and specificity of CTRP-1, TNF-α, and IL-10 at their optimal cut-off values for distinguishing between different periodontal conditions. In the comparison between the H and G groups and between the H and P groups, all biomarkers and their combinations demonstrated excellent discrimination (AUC = 0.974-1). In contrast, in the comparison between the G and P groups, the performance of all biomarkers was comparatively lower (AUC = 0.762–0.911).

**Table 5:** Diagnostic potential of cytokines

	AUC	%95 CI	Sensitivity(%)	Specificity(%)	Cut-Off	*p*
**Healthy vs. Gingivitis**						
CTRP-1 (pg/30sn)	0.974	0.881–0.999	91.3	96	> 14.26	**< 0.001**
TNF-α (pg/30sn)	0.944	0.838–0.990	91.3	100	> 0.32	**< 0.001**
IL-10 (pg/30sn)	1	0.926-1	100	100	> 1.14	**< 0.001**
CTRP-1 + TNF-α	1	0.926-1	100	100	≤ 0	**< 0.001**
CTRP-1 + IL-10	1	0.926-1	100	100	≤ 0	**< 0.001**
TNF-α + IL-10	1	0.926-1	100	100	≤ 0	**< 0.001**
CTRP-1 + TNF-α + IL-10	1	0.926-1	100	100	≤ 0	**< 0.001**
**Healthy vs. Periodontitis**						
CTRP-1 (pg/30sn)	0.998	0.926-1	100	96	> 14.26	**< 0.001**
TNF-α (pg/30sn)	1	0.929-1	100	100	> 0.325	**< 0.001**
IL-10 (pg/30sn)	1	0.929-1	100	100	> 1.14	**< 0.001**
CTRP-1 + TNF-α	1	0.929-1	100	100	≤ 0	**< 0.001**
CTRP-1 + IL-10	1	0.929-1	100	100	≤ 0	**< 0.001**
TNF-α + IL-10	1	0.929-1	100	100	≤ 0	**< 0.001**
CTRP-1 + TNF-α + IL-10	1	0.929-1	100	100	≤ 0	**< 0.001**
**Gingivitis vs. Periodontitis**						
CTRP-1 (pg/30sn)	0.762	0.617–0.873	64	91.3	> 20.46	**0.002**
TNF-α (pg/30sn)	0.817	0.679–0.914	72	82.6	> 0.552	**< 0.001**
IL-10 (pg/30sn)	0.911	0.793–0.974	80	91.3	> 2.22	**< 0.001**
CTRP-1 + TNF-α	0.831	0.695–0.924	72	100	≤ 0.331	**< 0.001**
CTRP-1 + IL-10	0.910	0.791–0.973	80	91.3	≤ 0.341	**< 0.001**
TNF-α + IL-10	0.913	0.795–0.975	84	91.3	≤ 0.424	**< 0.001**
CTRP-1 + TNF-α + IL-10	0.913	0.795–0.975	84	91.3	≤ 0.431	**< 0.001**

Abbreviations: CTRP-1: Complement-C1q tumor necrosis factor-related protein 1; TNF-α: Tumor necrosis factor-alpha; IL-10: Interleukin-10; pg: picograms; AUC: area under the curve; CI: confidence interval of 95%. The cut-off values were calculated according to the Youden’s J index. ***p* < 0.05 Statistical differences are marked in bold**

## Discussion

To our knowledge, no previous study has reported the detection of CTRP-1 in the GCF of both periodontally healthy and diseased individuals. This study evaluated the levels of GCF CTRP-1, TNF-α, and IL-10 in the H, G, and P groups, along with their variations in response to NSPT. The key findings were as follows: (1) Increased levels of GCF CTRP-1 showed a significant association with both the G and P groups; (2) NSPT contributed to a significant reduction in the levels of all biomarkers in patients within the G and P groups; and (3) GCF CTRP-1 may serve as a possible biomarker for the detection of periodontal diseases.

CTRP-1 has been implicated in the pathophysiology of various systemic diseases beyond periodontal inflammation, underscoring its broader role in immunometabolic regulation. Elevated circulating levels of CTRP-1 have been reported in individuals with type II diabetes mellitus, obesity, atherosclerosis, hypertension, where it is thought to modulate immune and metabolic responses through cytokine regulation and other signaling pathways [13, 14, 28, 29]. In metabolic disorders such as diabetes and obesity, increased CTRP-1 levels are positively associated with insulin resistance, dyslipidemia, and low-grade chronic inflammation [13, 28]. In cardiovascular diseases, CTRP-1 has been linked to endothelial dysfunction, arterial stiffness, and the progression of atherosclerosis [14]. Similarly, elevated CTRP-1 levels in patients with hypertension suggest a role in vascular dysfunction and inflammatory processes that contribute to the pathogenesis of this condition [29]. Importantly, its association with systemic diseases other than periodontal conditions has been consistently demonstrated in clinical and experimental studies. These observations collectively indicate that CTRP-1 may function as an immune modulator involved in both local and systemic inflammatory processes.

The significantly higher levels of CTRP-1 observed in individuals with periodontal disease, as compared to healthy individuals, suggest its potential involvement in the pathogenesis of periodontal disease. While no prior studies have specifically investigated CTRP-1 in periodontal disease, its role as a modulator of inflammation- through its influence on pro-inflammatory molecules such as TNF-α and IL-6- has been well established in other inflammatory conditions [11, 12, 30]. The increased levels of CTRP-1 in sites affected by periodontal disease may reflect a compensatory mechanism in response to increased inflammatory burden. Moreover, the post-treatment decrease in CTRP-1 levels further supports its role in periodontal inflammation and the response to therapy. The observed findings align with previous research on the other adipokines, reinforcing the concept that metabolic mediators influence periodontal disease progression [31, 32]. Adipokines, such as leptin, visfatin, resistin, and adiponectin, have been extensively studied in this context, with substantial evidence supporting their involvement in inflammatory and immune responses. Previous studies have demonstrated that resistin and visfatin levels in GCF were elevated in patients with periodontitis compared to healthy controls, and decreased following NSPT [6, 9, 31]. Conversely, lower GCF levels of adiponectin and leptin have been observed in periodontitis patients, with an increase post-NSPT, suggesting a dynamic regulation of adipokines in response to periodontal inflammation and treatment [6, 32].

This study yielded several anticipated findings. The elevated levels of TNF-α observed in patients with periodontitis corroborate its well-established role in periodontal inflammation, bone resorption, and connective tissue destruction [16, 33, 34]. Furthermore, the subsequent reduction in TNF-α following NSPT is consistent with previous studies demonstrating the efficacy of periodontal therapy in diminishing inflammatory burden [35–37]. Conversely, IL-10, an anti-inflammatory cytokine known for its regulatory effects on immune responses, exhibited elevated levels in periodontitis patients at baseline. This finding may indicate an attempt by the host to counteract the excessive inflammation. The presence of IL-10 in GCF and its variations with disease progression and treatment were also expected, given its established role as key regulator of immune responses. Although its primary function is to suppress pro-inflammatory responses and promote tissue hemostasis, its role in periodontitis remains controversial. While our findings align with research suggesting that IL-10 act as a countermeasure against inflammation [20, 36, 38], some studies have reported inconsistent findings, with lower IL-10 levels observed in severe periodontitis cases, suggesting that its expression may be influenced by the severity of the disease and immune responses [39, 40]. Interestingly, our study observed a reduction in IL-10 levels after NSPT, possibly reflecting an immune shift as inflammation was resolved. This finding is consistent with reports indicating a reduction in IL-10 following treatment [41, 42]. However, other studies have reported conflicting results, with some documenting increased IL-10 levels at periodontal disease sites [43, 44], while others found no significant change after NSPT [45]. These discrepancies highlight the versatile role of IL-10 in periodontal disease and need for further investigation.

The present study underscores the high sensitivity of CTRP-1 in differentiating individuals with periodontal disease from healthy individuals, demonstrating its strong diagnostic accuracy. Logistic regression analysis further confirmed that CTRP-1, TNF-α, and IL-10 were significantly associated with periodontitis, even after adjusting for possible confounders, such as age and BMI; however, it is important to consider the potential influence of the mean age difference between the study groups on these outcomes. Age has a well-established impact on systemic inflammatory responses, including modulation of cytokine production [46]. Although age was statistically adjusted for in the regression models, age-related alterations in immune function and cytokine expression could still have contributed to the observed differences. Therefore, future studies with age-matched cohorts are recommended to better isolate the independent role of CTRP-1 and inflammatory markers in periodontitis.

ROC curve analysis revealed that combinations of CTRP-1, TNF-α, and IL-10 exhibited excellent diagnostic accuracy in differentiating periodontitis and gingivitis from a healthy condition. However, due to the lack of prior research on the AUC values of CTRP-1, direct comparisons with previous studies were not possible. Despite its promising diagnostic potential, the utility of CTRP-1 as a standalone biomarker remains limited due to the multifactorial nature of periodontal disease pathogenesis and should therefore be interpreted with caution. Nevertheless, the findings regarding TNF-α and IL-10 are consistent with those of prior research on inflammatory biomarkers in periodontal disease. ROC analyses have shown that both TNF-α and IL-10 exhibit high AUC value, supporting their utility as biomarkers in periodontal disease status [20, 31, 47].

The concentration of the GCF is directly affected by its volume. The increased GCF volume, commonly observed in periodontal disease, may lead to the dilution of cytokine concentrations. Consequently, this study assessed the total amount per sample to mitigate the effects of volume variation [20, 47].

The primary limitation of this clinical study is its focus on evaluating biomarker levels in patients with SIIIGB periodontitis, without assessing potential variations across different disease stages and grades. Given that the progression rate and severity of periodontitis can influence inflammatory responses and biomarker levels, future studies are required to investigate these factors to supply a more comprehensive understanding of the disease process.

## Conclusion

Considering the limitations of this study, CTRP-1 has the potential to serve as biomarker for monitoring periodontal disease. TNF-α and IL-10 levels are also correlated with periodontal status, with IL-10 possibly reflecting a regulatory response. Further research is needed to explore CTRP-1’s role in pathogenesis and its therapeutic potential.

## Data Availability

No datasets were generated or analysed during the current study.
